# Supplementary material for: Speedy bearings to slacked steering: Mapping the navigation patterns and motions of Viking voyages
Source: PLoS One. 2023 Nov 22;18(11):e0293816. doi: 10.1371/journal.pone.0293816 (PMC10664959; doi:10.1371/journal.pone.0293816)
Supplement: Attachment — Submitted filename: +VikingPolSunCloud_PLoS-One-response-2.docx [file pone.0293816.s001.docx]

**Point-by-Point Response to the Comments of Reviewer 3**

We thank Reviewer 3 for her/his comments. Our manuscript was revised on the basis of the reports of two new Reviewers (3, 4). All changes suggested/inspired by Reviewer 3 and Reviewer 4 are marked with red and violet, respectively. Below is our response to the comments of Reviewer 3.

First of all, we would like to emphasize our disappointment due to the immensely negative opinion of Reviewer 3 about our manuscript, who focused almost exclusively on the archeological evidences and cultural aspects of Viking navigation. Her/his report contradicts those of Reviewers 1, 2 and 4, who were quite satisfied with the 1st revision of our manuscript.

The comments, concerns, criticisms and objections of Reviewer 3 can be classified into three groups:

A) Constructive comments which help the amendment of our manuscript.

B) Valueless criticisms without justifications, explanations or concretes.

C) Unsound comments, the indefensibility of which is explained in our answers.

The constructive comments (group A) are the following:

**A1) Reviewer 3 wrote**: *- The time period referred to as 'Viking' extends well beyond the historically accepted age; even in previous publications by the authors it is stated to have been c.300 years but here it is c.500 into the 1400. The reference used for this dating, though relevant to this discussion, is not reflective of the historiography of the end of the Viking age in c. 1066. I would suggest major revision of this time period and a more comprehensive 'background' and historical contexts especially when so closely relating this HYPOTHETICAL practice to the historic culture. This also has to take into account sailing technology and by 1400 the construction of ships and maritime technology in Northern Europe is significantly different. As such the conclusions drawn and the framework with which the experimentation has been undertake has no basis and thus analysis has not been undertaken to a sufficient standard.*

**Answer**: As suggested by Reviewer 3, the 1st sentence of the Abstract is changed as follows:

”Viking sailors ruled the North Atlantic Ocean for about three hundred years.”

Similarly, the 1st sentence of the Introduction is changed as follows:

”Viking sailors ruled the North Atlantic Ocean for about three hundred years (between *Anno Domini* 900 and 1200) [1].”

Note, however, that this simple change/correction does not influence at all any of our conclusions and the framework with which our experimentation has been undertaken.

**A2) Reviewer 3 wrote**: *- The use of the phrase 'lazy' I really struggle with; certainly any sailing of this distance would not be done lazily and the societal connotations that come with the use of this phrase are inappropriate in this context.*

**Answer**: In our paper, the attribute ‘diligent’ was used as a shorter synonym of ‘hard-working’, which was used to name Viking navigators who are navigating with periodicity 1 h ≤ Δ*t* ≤ 2 h. In order to be more precise, in our 2nd revised manuscript we changed ‘diligent’ and ‘hard-working’ to ‘often-navigating’, while ‘medium-working’ was replaced by ‘medium-navigating’, and ‘lazy’ was changed to ‘seldom-navigating’.

**A3) Reviewer 3 wrote**: *- The fact that this is not based upon something widely accepted within archaeology needs to be addressed more clearly and to present it as 'corroborated' in such a manner is another significant flaw and is deceptive.*

**Answer**: As suggested by Reviewer 3, in the revised Abstract we wrote:

”Many details of the hypothetical SPVN have been thoroughly revealed in psychophysical laboratory and planetarium experiments.”

Furthermore, in the revised Introduction we wrote:

”This hypothetical orientation method is named ’sky-polarimetric Viking navigation’ (SPVN). According to the hypothesis, sunstones might have been dichroic or birefringent crystals, such as tourmaline, cordierite or calcite, functioning similarly as linear polarization analyzers [12, 13, 14, 15]. We emphasize, however, that in spite of the wide-spread hypothesis of SPVN, it is not based upon archeological artefacts or written documents.”

**A4) Reviewer 3 wrote**: *- This research would significantly benefit from interacting more with literature of computer simulated experimental archaeology and present itself more explicitly as hypothetical; seeing if there is any advantage to be gained in using the navigational strategies as suggested against simulations of accepted methods of navigation understood to be used by Viking sailors rather than 'lazyness'.*

**Answer**:

**Ad 1)** The issue of ‘lazyness’ is addressed in point A2.

**Ad 2)** In the revised Abstract we wrote:

”What was so far lacking in this experimental and computational archeological approach is the study of the success of SVN and a combined navigation using solar cues in sunshine (SVN) and sky polarization at invisible Sun (SPVN), the latter being the most realistic method.”

**Ad 3)** In the revised Introduction we wrote:

”What was so far lacking in this experimental-computational archeological approach is the study of the success of SVN and a combined navigation method using solar cues in sunshine (SVN) and sky polarization when the Sun is invisible (SPVN).”

**A5) Reviewer 3 wrote**: *I have questions over its practicality onboard a moving ship and in a greater range of environmental factors (e.g. rain or if water has effect).*

**Answer**: To the revised Discussion we added the following:

We admit that the method of SPVN is rather complex, and nobody knows whether it has been really used by Viking sailors. Its practicality onboard a moving-waggling ship could be questioned, especially under rainy and/or windy conditions. The effects of these disturbing environmental factors on the success *S* of Viking navigation are unknown and practically cannot be determined. It is, however, sure that they more or less reduce the *S*.

**A6) Reviewer 3 wrote**: *The paper disregards a number of other factors of sail and also other navigational traditions, that the sun was not the primary tool for navigation but also following bird migratory patterns, the prevailing and seasonal winds, star and moon cycles and other means of navigating that are recorded with oral tradition and have also been tested in experimental practical's and found to be effective. Ignoring other factors and fundamentally the process of sailing renders the findings and conclusion of this difficult to accept as there are significant environmental and cultural inputs into the calculations missing other that 'laziness' or there being clouds.*

**Answer**: To the beginning of the revised Discussion we added the following:

Although the Sun and possibly the sky polarization were surely not the only tools for Viking navigation, the aim of this work is to estimate the success of four possible navigation methods using solely the Sun and/or sky polarization without other information. Therefore, our modelling disregarded a number of other factors of sail and also certain navigational traditions, such as following the migratory patterns of birds and whales, the prevailing and seasonal winds, stars and moon cycles and other means of navigating that are recorded with oral tradition and have also been tested experimentally and found to be effective [1, 2, 3, 4, 5, 11, 12].

**A7) Reviewer 3 wrote**: *- There is a premise that if the sun is not visible then the ship would stop and no navigation would take place, there are a range of issues with this as again aforementioned if considering partial use of the sun-stone as a hypothetical practice then it would have been but part of an arsenal of tools and sailing would not simply 'stop' as it would likely mean loss and death of the crew.*

**Answer**: Nobody knows exactly the arsenal of navigational tools Vikings used, and there is no information about the sailing behaviour of Vikings when they did not see the Sun. In our paper we do not want to speculate about this issue. Therefore, to the paragraph describing Strategy 4 we added only the following:

”**Strategy 4 (SVN with stop)**: The navigator did not use sky polarization, thus navigation was possible only when the Sun was visible (calculated with the same method as described at Strategy 2), and the sailing stopped when the Sun was invisible. This method uses only step 4 of SPVN. Although it is also unknown what Vikings did when they did not see the Sun, the aim of the introduction of this strategy into our study is to determine the effect of ship’s stop at occluded Sun on the success of SVN.”

**A8) Reviewer 3 wrote**: *- In explanation it would benefit from a diagram; discussion of scale would also show how these process of navigation are expected to take place.*

**Answer**: As suggested by Reviewer 3, we added the new Figure 1 showing the four steps of sky-polarimetric Viking navigation. In the revised Introduction we wrote:

“The SPVN has four steps [16, 17, 18] (Fig. 1): 1) Determination of orientations O1 and O2 perpendicular to the directions of polarization of skylight in two celestial points C1 and C2 with the use of sunstones (Fig. 1A). 2) Determination of the position P of invisible (cloud-occluded) Sun coinciding with the intersection P of the two great circles passing through C1 and C2 and parallel to O1 and O2 (Fig. 1B). 3) Determination of the elevation angle θ of P (invisible Sun) (Fig. 1C). 4) In the knowledge of the position of P (i.e. θ and the azimuth direction of P), determination of the geographical north with the use of the sun-compass and a shadow-stick (Fig. 1D).”

**Fig. 1. The four steps of sky-polarimetric Viking navigation [16, 17, 18].** (A) In the 1. step, the Viking navigator looked at two points of the polarized sky through two sunstones. Rotating both sunstones, he determined the two directions pointing toward the Sun. These directions were scratched onto the sunstones as red arrows. (B) In the 2. step, he estimated the intersection of the two celestial great circles crossing the red arrows. This intersection coincides with the position of the occluded Sun. (C) In the 3. step, he measured the elevation θ (calculated in fist and finger) of the estimated position of the occluded Sun (θ = 3 fists + 0 finger in C). (D) In the 4. step, he placed the hole (now 3.) of the shadow-stick corresponding to θ onto the tip of the vertical gnomon of the sun-compass while orienting the long axis of the shadow-stick toward the estimated position of the occluded Sun. He turned the horizontal disc of the sun-compass around the gnomon until the mirror-symmetric hyperbolic shadow curve scratched onto the disc hit the end of the shadow-stick resting on the disc. Then the symmetry axis of the shadow curve (marked by an arrow scratched into the disc) pointed toward the geographical North.

The valueless criticisms (group B) are the following:

**B1) Reviewer 3 wrote**: *That claims and findings to its historic context: There are claims within this paper that are unsubstantiated and culturally loaded phrases that are objectionably used.*

**Answer**: Reviewer 3 should explicitly explain which claims of ours are unsubstantiated, and which culturally loaded phrases of ours are objectionably used, and why. Until this detailed explanation is not performed, this criticism is valueless.

**B2) Reviewer 3 wrote**: *- Using the effort of 'Vikings' as a scale in which to draw statistical hypothesis against is not technically sound. The suggestions and presumptions of it renders the findings redundant and thus I can not see this under its current format or hypothesis being suitable for publication. The conclusions are thus not presented in an appropriate fashion.*

**Answer**: This criticism is again valueless, because it is too general and contains no any concrete.

- Reviewer 3 states here that our approach is not technically sound, but she/he does not explain why.

- She/he claims without any justification that our findings are redundant.

- She/he asserts that our conclusions are not presented in an appropriate fashion, but she/he does not explain the right/desired fashion of presentation.

**B3) Reviewer 3 wrote**: *That the methodology has not been expressed clearly.*

*The paper is confusing in description of its methods and findings, in part because of the use of an effort scale of measurement that is not adequality substantiated.*

**Answer**: Reviewer 3 should communicate explicitly which details of our methodology and findings are not clearly described in our paper. Until this is not correctly explained, this criticism is valueless and cannot help the improvement of our paper. Note that Reviewers 1, 2 and 3 were satisfied with the description of our methods and findings.

**B4) Reviewer 3 wrote**: *- It would significantly benefit and enrich finding in considering other variables to prove the advantage of using the polarization and if it leads to navigational success (this also needs clearer definition in the introduction).*

**Answer**: This is again a valueless comment, until Reviewer 3 does not say explicitly which ‘other variables’ should still be taken into account and incorporated into the studied navigational methods.

Furthermore, what should be clearer defined in the Introduction? Please be more specific and more explanatory.

**B5) Reviewer 3 wrote**: *This paper struggles with some fundamental principles, the calculations under the 4 navigational strategies are not based on cultural practice and the hypothesis and methodology are thus missing meaningful components and renders the conclusion unacceptable. This paper would not make a contribution to studies. Although the mathematical work appears sound the argument of this paper falls beyond the scope of its calculations, owing to its limited range of factors. Even with major revision it is in the principle premise of this paper and ignorance of a range of other factors in its calculations and ignorance of the process of sailing itself, that issue is found and thus I have suggested rejection of this paper.*

**Answer**: This valueless list is nothing else as a summary of the earlier offensive concerns, unsound objections and erroneous statements of Reviewer 3 without any explanation and justification. Above we answered in detail also these comments.

The unsound comments (group C) are the following:

**C1) Reviewer 3 wrote**: *I have the following concerns about this article: That the research is not sufficiently original - There are already a range of publications that share significant similarities in the content and language of this paper and there are concerns about dual publication and the cross over of materials with some paragraphs being very similar to previous works of the authors that falls within the scope of self-plagiarism.*

**Answer**: This concern is unsound. The results presented in our present manuscript are original and obviously based on our earlier results cited appropriately. Our earlier similar publications have dealt with different aspects of sky-polarimetric Viking navigation (SPVN). In these papers we have always revealed new details of SPVN, like in our present manuscript. Since the main subject of investigation (topic) of these papers is SPVN, it is quite understandable that their contents (e.g. literature review, introduction, method) and language (e.g. technical terms) are similar. The lack of such similarity would be rather surprising. In spite of this similarity, there is not any dual publication or self-plagiarism. With this concern, Reviewer 3 practically frames us with dual publication and/or self-plagiarism! If yes, she/he should explicitly evidence which parts of our present manuscript are dual-publiced/self-plagiarised.

The Introduction of our present paper describes clearly the purpose and novelty of our study:

”What were so far lacking in this experimental-computational archeological approach are the following:

- The study of the success of SVN and a combined navigation method using solar cues in sunshine (SVN) and sky polarization when the Sun is invisible (SPVN). Obviously, in sunshine it is not worth using SPVN instead of the much simpler SVN. Since during a several-week voyage both sunny and cloudy skies occur frequently, the use of the combined SVN-SPVN method is more realistic than the mere use of the four-step complex SPVN having four error functions contrary to the one-step SVN.
- The purpose of this work is to determine the success of the sole SVN and the combined SVN-SPVN relative to the mere SPVN. We investigated four different navigation strategies/methods: 1) SPVN, using only sky polarization. 2) SVN or SPVN, using SVN in sunshine and SPVN under cloudy conditions. 3) SVN without stop, continuing sailing when the Sun is invisible. 4) SVN with stop, ceasing sailing when the Sun is invisible. The success of these four navigation strategies was determined for three different navigator types (determining the intended sailing direction with large, medium or small frequencies) at spring equinox and summer solstice, with and without night sailing. We demonstrate here that if the navigators’ aim is to maximize the sailing success, they have to choose different navigation strategies depending on their navigation frequency.
- Compared to earlier studies that considered only SPVN [30, 31], the uniqueness of our present work is that we investigate here not only SPVN, but also two further navigation methods using the sun-compass only in sunshine and continuing/ceasing sailing when the Sun is not visible. Furthermore, we also studied the fourth, combined method, which uses only the sun-compass when the Sun shines, while under a cloudy sky it analyses the sky polarization with a calcite sunstone in order to be able to use the sun-compass. Further novelty is that contrary to earlier studies, in this work we compare the navigation successes of often-, medium- and seldom-navigating sailors for nine different combinations of date (equinox, solstice) and night sailing (cease or continuation of sailing at night) versus the four navigation methods.”

Furthermore, the subsection ’Novelties of our simulations’ deals also with the novelties of our present simulations:

**”**In our present simulations, we improved our earlier approach of simulated voyages using SPVN [31]. Here we describe in detail only the new or most important issues of simulation, especially the four different navigational strategies Viking sailors could apply. Our main aim was to find the best strategy ensuring the highest navigational success *S*. The simulated Viking voyages from Norway to Greenland ran along the 60° 21' 55'' latitude. The parameters of simulations were the sailing date (spring equinox: 21 March, or summer solstice: 21 June, night sailing (sailing or not sailing at night), solar elevation, ship’s speed, start time of diurnal sailing, north error, visibility distance of Greenland’s southern coastline, navigation periodicity (0.5 h ≤ Δ*t* ≤ 6 h), dominance (-1 ≤ *m* ≤ 1) and changeability (1 ≤ σ ≤ 4) of cloudiness. The settings of these parameters and all other details of the simulation of Viking voyages are described in [31].”

With the above arguments, we deny the rude prosecution of dual publication/self-plagiarism!

**C2) Reviewer 3 wrote**: *- The paper claims to be 'archaeologically corroborated' however this is not substantiated and is rather based upon outdated and heavily criticised research from the archaeological discipline. In previous papers by the authors it is made clear that this is fundamentally a calculation of experimental archaeology, but that some have take the premise further. There is currently no securely dated or contemporary Viking artefact to base these calculations off but that there is lore or myth that such sun stones were used - it has been interesting to read research into if this is a viable mode of navigation however, such research has already been published and I do not find that the results of this paper to add further to these explorations.*

**Answer**:

**Ad 1)** The attribute ‘archeological’ occurs only once in the Introduction as follows:

”It is an archeologically corroborated fact that Viking navigators used a sun-compass by which they could determine the direction of the geographical north in sunshine [7, 8, 9].”

7. Thirslund S. 1991; A presumed sun compass from Narsarsuaq. In The Church topography of the eastern settlement and the excavation of the Benedictine convent at Narsarsuaq in the Uunartoq Fjord (ed Vebæk CL), pp. 65-71. Museum Tusculanum Press: Copenhagen, Denmark.

8. Thirslund S. The discovery of an early bearing-dial - Further investigations. Journal of Navigation 1993; 46: 33-48.

9. Filipowiak W. How Vikings crossed the North Atlantic? The reinterpretation of ‘sun compasses’ - Narsarsuaq, Wolin, Truso. International Journal of Nautical Archaeology 2020; 49: 318-328 (doi: 10.1111/1095-9270.12426).

This statement is correct and supported by references [7, 8, 9].

**Ad 2)** Reviewer 3 errs, because we did not state at all that all aspects and hypotheses of our paper would be archeologically corroborated. We stated here only that the existence of Viking sun-compasses is archeologically corroborated.

**Ad 3)** We never (in no papers of ours) stated that there would be archeological artefacts or written documents of the hypothetic Viking sunstones and their use to analyse sky polarization for navigational purposes. If Reviewer 3 reads our articles dealing with sky-polarimetric Viking navigation (SPVN), it should be clear that the four steps of SPVN studied by us are hypothetical, and are based on the assumptions published earlier by others. We followed these assumptions and using psychophysical experiments, sky-polarimetric measurements and computer modelling, we investigated in detail all aspects of SPVN in order to test the reliability of this hypothetical navigation method.

**Ad 4)** In our answer C1 we clearly explained the noveltly of the results of our present paper.

**Ad 5)** From the totally negative opinion, offensive comments and unsound criticisms of Revierwer 3 – who focuses almost exclusively the archeological aspects and evidences of SPVN – it is evident for us that she/he does not like/accept this wide-spread hypothesis of SPVN, and therefore she/he is quite prejudiced. This scientifically unacceptable prejudice, unfortunately, hinders her/him to give an objective and correct review.

**Point-by-Point Response to the Comments of Reviewer 4**

We thank Reviewer 4 for her/his constructive and valuable comments. Our manuscript was revised on the basis of the reports of two new Reviewers (3, 4). All changes suggested/inspired by Reviewer 3 and Reviewer 4 are marked with red and violet, respectively. Below is our response to the comments of Reviewer 4.

**Reviewer 4 wrote**: *The manuscript is technically and scientifically well researched and findings of the research are adequately supported by the tables and figures which enumerate the navigational strategies and successes of the Vikings. The research methodically traces the rhythms of Viking voyages radiating from the coastal sites to the high sea and how within themselves they differed in negotiating the safe passage between Norway and Greenland during the Equinox and Solstice.*

**Answer**: We are grateful to Reviewer 4 for her/his positive opinion. :-)

**Reviewer 4 wrote**: *In one of the Indian Ocean navigational strategies, the Polaris and the moon were used as an indicator of time at night. In this strategy, the lunar calendar was stringently followed where the movement of the moon in each constellation and zodiac sign was primarily taken into account. To determine the direction, some approximate calculations (Zam) were made, keeping before them the apparent motion of sun and North Star (Daru/Dhruv). Moreover, after knowing the angular distance of the sun during noon and its shifts, the navigator could then calculate and note the latitude degrees in their logs accordingly. During the night the rising time of the North Star and the position of a cluster of other stars were important pointers for steering. It was known that there would be delays of some minutes at the time of rise of stars. This information was used in gauging a particular direction. The units used for such celestial navigation was believed to follow cosmic laws and be attuned to energies of the universe. Being guided by the monsoon winds the Malam (captain) was required to steer to sail north or south, as suitable, to bring Polaris to the altitude of the home port. There after the vessel needed to steer the angular distance, or merely sail down the latitude, chiefly keeping Polaris at a constant angle. This calculation of distance was a key to the success of the voyage as the slightest mistake in calculation would mar the entire endeavor.*

*Based upon the above Indian maritime sailing knowledge of celestial navigation my question to the authors is regarding the usage of any such techniques by the Vikings in case of the night sailing and navigational strategies. If yes then please incorporate such information for it would be intriguing to know how did the Vikings steer in the night. Did they simply calculate the latitude of Polaris as they progressed in the night sailing?*

*What I understand through authors' research is the Vikings slowed down in the night. I want authors to quickly rethink or tweak on this point, especially, considering the hardworking sailors and their approach.*

**Answer**: Inspired by the above useful comment of Reviewer 4, to the beginning of the revised ‘Discussion’ section we added the following:

In one of the navigational strategies of sailors crossing the Indian Ocean, at night the Polaris and/or the Moon served as time indicator (personal communication of Reviewer 4): ”*In this strategy, the lunar calendar was stringently followed where the movement of the moon in each constellation and zodiac sign was primarily taken into account. To determine the direction, some approximate calculations (Zam) were made, keeping before them the apparent motion of the Sun and North Star (Daru/Dhruv). Moreover, after knowing the angular distance of the Sun during noon and its shifts, the navigator could then calculate and note the latitude degrees in their logs accordingly. During the night the rising time of the North Star and the position of a cluster of other stars were important pointers for steering. It was known that there would be delays of some minutes at the time of the rise of stars. This information was used in gauging a particular direction. The units used for such celestial navigation was believed to follow cosmic laws and be attuned to energies of the universe. Being guided by the monsoon winds the Malam (captain) was required to steer to sail north or south, as suitable, to bring Polaris to the altitude of the home port. Thereafter the vessel needed to steer the angular distance, or merely sail down the latitude, chiefly keeping Polaris at a constant angle. This calculation of distance was a key to the success of the voyage as the slightest mistake in calculation would mar the entire endeavor.*”

Although the above Indian maritime sailing knowledge of celestial navigation could have been very helpful also for Viking navigators, it is unknown whether they used any such or similar techniques during their night sailing. In principle, they could have simply calculated the latitude of Polaris as they progressed in the night sailing. However, unfortunately nobody knows how the Vikings steered in the night. Since we did not want to speculate on this, in our navigation model we assumed the simplest possibility of night sailing: at night the Viking ship either continued its course, or stopped until sunrise. In the latter case the average sailing speed is underestimated. The effect of this on the sailing success was, however, studied by comparison to the case when at night Vikings continued their course.

In order to avoid plagiarism, the comment of Reviewer 4 was cited between quotation marks.

- Is this acceptable by Reviewer 4?

- Could Reviewer 4 write her/his name in order to specify it in the above paragraph?

- Or should we delete this comment between the quotation marks?

- Could Reviewer 4 write us a reference dealing with the above-mentioned Indian maritime nocturnal celestial navigation?
