# Supplementary material for: Speedy bearings to slacked steering: Mapping the navigation patterns and motions of Viking voyages
Source: PLoS One. 2023 Nov 22;18(11):e0293816. doi: 10.1371/journal.pone.0293816 (PMC10664959; doi:10.1371/journal.pone.0293816)
Supplement: Attachment — Submitted filename: +VikingPolSunCloud_PLoS-One-response-3.docx [file pone.0293816.s002.docx]

**Point-by-Point Response to the Comments of the**

**Academic Editor, Zahid Mahmood Jehangiri**

**Academic Editor wrote**: *In this round, the review was made by a total of 4 reviewers. Out of them Reviewers 1 to 3 are new reviewers. While, Reviewer 4 was the previous reviewer. Seeing the reviews of the reviewer, I propose to authors to strictly follow the followings.*

**Answer**: We thank for the valuable and constructive comments of the Academic Editor, Zahid Mahmood Jehangiri. Our manuscript was revised on the basis of the reports of three new Reviewers (1, 2, 3) and one old Reviewer (4). All changes suggested by Reviewer 1, Reviewer 2, Reviewer 3 and Reviewer 4 are marked with red, blue, orange and violet, respectively. Below is our response to the comments of the Academic Editor.

**Academic Editor wrote**: *1. Please revise the title of this manuscript. It is very lengthy.*

**Answer**: The shortened new title suggested by Reviewer 4 is:

Speedy Bearings to Slacked Steering: Mapping the Navigation Patterns and Motions of Viking Voyages

**Academic Editor wrote**: *2. Authors must include a pseudocode of their developed method.*

**Answer**: As suggested by Reviewer 2, to demonstrate qualitatively our developed computation/simulation method, the following new Figure 2 is inserted into subsection ‘2.2. Novelties of simulations’ of the revised ‘2. Methods’ section:

**Fig. 2. Block diagram of our developed computation/simulation of Viking navigation.**

**Academic Editor wrote**: *3. Authors must improve the resolution of all figures as per the*

*reviewers concerns.*

*5. Authors must improve the resolution of figures provided in the manuscript.*

**Answer**: The original resolution of the submitted tiff-format figures was high enough, but during the pdf-conversion the resolution became reduced. In the final published version all figures will have high resolution.

**Academic Editor wrote**: *4. Authors must include a mathematical analysis in this manuscript.*

**Answer**: Under ‘**mathematical** analysis’ the Editor might have thought **statistical** analysis. In our paper there is no statistical analysis. The meaning of navigation success *S* (%) = *N*_successful_/(*N*_successful_ + *N*_unsuccessful_) in Figures 1-10 is the proportion of the number of successful (*N*_successful_) and the number of total (*N*_successful_ + *N*_unsuccessful_) simulated Viking voyages, rather than statistical significance. The *S*-values can be considered as the simplest statistics.

**Academic Editor wrote**: *6. Authors must include the latest references in revised version of the manuscript.*

**Answer**: The research field of Viking navigation develops only slowly due to the only sporadic new data, information and archaeological findings. Since there are only a few scientists who study this subject, only very few publications appear yearly/decadely in this topic. This is the reason for the lack of many references from the last 5 years. Nevertheless, in the ‘Introduction’ section we cited the following 5 new, most recent references:

7. Vanin G. The beginning of celestial navigation. arXiv:2209.02371 2022. (https://doi.org/10.48550/arXiv.2209.02371).

13. Craecker-Dussart de C. Moyens d’orientation et de navigation des Vikings, marins accomplis en Atlantique Nord (fin VIII^e^–XI^e^ siècles). Le Moyen Age 2019; 3-4: 617-650 (doi: 10.3917/rma.253.0617).

38. Liu J, Zhao L, Qi B, Cheng J, Cai J. A new polar integrated alignment algorithm with the aids of DVL and the improved polarized-light navigation. Proc. 5th Int. Symp. Auto. Syst. (ISAS) 2022; 1-6 (doi.org/10.1109/ISAS55863.2022.9757316).

39. Courtier G, Lapray P-J, Adam R, Changey S, Lauffenburger J-P. Ground vehicle navigation based on the skylight polarization. IEEE/ION Position, Location and Navigation Symposium (PLANS) 2023: Monterey, United States. pp. 1373-1379 (doi: 10.1109/PLANS53410.2023.10140044).

40. Li Q, Dong L, Hu Y, Hao Q, Wang W, Cao J, Cheng Y. Polarimetry for bionic geolocation and navigation applications: a review. Remote Sensing 2023; 15: 3518 (doi: 10.3390/rs15143518).

Thus, together with the above 5 new references, in our revised manuscript there are 9 references from the last five (2019-2023) years including the followings:

10. Filipowiak W. How Vikings crossed the North Atlantic? The reinterpretation of ‘sun compasses’ - Narsarsuaq, Wolin, Truso. International Journal of Nautical Archaeology 2020; 49: 318-328 (doi: 10.1111/1095-9270.12426).

33. Takács P, Száz D, Pereszlényi Á, Horváth G. Sensitivity and robustness of sky-polarimetric Viking navigation: Sailing success is most sensitive to night sailing, navigation periodicity and sailing date, but robust against weather conditions. 2022; PLoS One 17: e0262762 (htpps://doi.org/10.1371/journal.pone.0262762)

36. Jin H, Wang X, Fan Z, Pan N. Linear solution method of solar position for polarized light navigation. IEEE Sensors Journal 2021; 21: 15042-15052 (doi: 10.1109/jsen.2021.3074416).

37. Dou Q, Du T, Qiu Z, Wang S, Yang J. An adaptive anti-disturbance navigation method for polarized skylight-based autonomous integrated navigation system. Measurement 2022; 202: 111847 (doi: 10.1016/j.measurement.2022.111847).

**If Reviewer 3 writes me the bibliographic data of such papers from 2019-2023, I shall add them to the reference list and cite them in our paper.**

**Point-by-Point Response to the Comments of the Reviewer 1**

We thank Reviewer 1 for her/his comments. Our manuscript was revised on the basis of the reports of three new Reviewers (1, 2, 3) and one old Reviewer (4). All changes suggested by Reviewer 1, Reviewer 2, Reviewer 3 and Reviewer 4 are marked with red, blue, orange and violet, respectively. Below is our response to the comments of Reviewer 1.

**Reviewer 1 wrote**: *My comment is to improve the quality of the figures and article structure. Figures are blur.*

**Answer**: The original resolution of the submitted tiff-format figures was high enough, but during the pdf-conversion the resolution became reduced. In the final published version all figures will have high resolution.

**Reviewer 1 wrote**: *Moreover, too lengthy table captions are usually not good in research articles.*

**Answer**: As suggested by Reviewer 1, from the caption of Table 1 the following text part was deleted:

(Eq+Ns, So+Ns, Eq-Ns, So-Ns, +Ns = Eq+Ns & So+Ns, -Ns = Eq-Ns & So-Ns, Eq = Eq+Ns & Eq-Ns, So = So+Ns & So-Ns, All = Eq+Ns & So+Ns & Eq-Ns & So-Ns, where & means union)

Similarly, from the caption of Table 4 the following text parts were deleted:

(often-navigating: determining the intended direction with periodicity Δ*t* = 1-2 h, medium-navigating: determining the intended direction with Δ*t* = 3-4 h, seldom-navigating: determining the intended direction with Δ*t* = 5-6 h) ... (1. Eq+Ns, 2. So+Ns, 3. Eq-Ns, 4. So-Ns, 5. +Ns = Eq+Ns & So+Ns, 6. -Ns = Eq-Ns & So-Ns, 7. Eq = Eq+Ns & Eq-Ns, 8. So = So+Ns & So-Ns, 9. All = Eq+Ns & So+Ns & Eq-Ns & So-Ns, where & means union)

Thus, the shortened captions for Tables 1 and 4 are the following:

**Table 1.** Success rank number *N* (I. = best with the largest navigation success *S*, II., III., IV. = worst with the smallest navigation success *S*) of the four (1, 2, 3, 4) navigation methods with averaged navigation success *S* in nine different cases for an often-navigating Viking determining the intended direction very frequently with periodicity Δ*t* = 1-2 hours. The higher the success *S*, the smaller the rank numeral (I., II., II., IV.). Eq: spring equinox, So: summer solstice, +Ns: sailing at night, -Ns: not sailing (stop) at night.

**Table 4.** Order numbers i. (= best sailor with the largest averaged navigation success *S*), ii. (= second sailor with smaller *S*), iii. (= worst sailor with the smallest *S*) of the three Viking navigator types in nine different cases versus the four navigation methods. The higher the success *S*, the smaller is the order number (i., ii., iii.). Eq: spring equinox, So: summer solstice, +Ns: sailing at night, -Ns: not sailing (stop) at night. The best order number i (and the *S*-value belonging to it) are in bold.

**Point-by-Point Response to the Comments of the Reviewer 2**

We thank Reviewer 2 for her/his valuable and constructive comments. Our manuscript was revised on the basis of the reports of three new Reviewers (1, 2, 3) and one old Reviewer (4). All changes suggested by Reviewer 1, Reviewer 2, Reviewer 3 and Reviewer 4 are marked with red, blue, orange and violet, respectively. Below is our response to the comments of Reviewer 2.

**Reviewer 2 wrote**: *1. Title of this manuscript is vague. Authors are advised to shorten the title.*

**Answer**: The shortened new title suggested by Reviewer 4 is:

Speedy Bearings to Slacked Steering: Mapping the Navigation Patterns and Motions of Viking Voyages

**Reviewer 2 wrote**: *3. Authors should number each section. Currently, it is missing.*

**Answer**: In the revised manuscript the sections are numbered.

**Reviewer 2 wrote**: *4. Please put a table for "Comparison of Viking navigators" and then explain. Current description is vague.*

**Answer**: Note that **Table 4 does compare the sailing success *S* of Viking navigators!** Thus, we can only slightly improve the presentation of the results summarized in Table 4 in the following ways:

1) In the revised manuscript the original term ‘strategy’ is replaced by ‘method’.

2) As requested by Reviewer 2, we added the following Table 5:

**Table 5.** The best navigator type (= having order number i. in Table 4 with the largest averaged navigation success *S* in the 9 different cases studied versus the four navigation methods. often: often-navigating sailor with periodicity Δ*t* = 1-2 h. medium: medium-navigating sailor with Δ*t* = 3-4 h. seldom: seldom-navigating sailor with Δ*t* = 5-6 h. Eq: spring equinox, So: summer solstice, +Ns: sailing at night, -Ns: not sailing (stop) at night. The most frequent navigator type (**often**-navigating) occuring in this table is in bold.

| cases | navigation method | | | |
| --- | --- | --- | --- | --- |
|  | 1 | 2 | 3 | 4 |
| 1. Eq+Ns | seldom | medium | **often** | **often** |
| 2. So+Ns | **often** | **often** | **often** | **often** |
| 3. Eq-Ns | **often** | **often** | **often** | **often** |
| 4. So-Ns | **often** | **often** | **often** | medium |
| 5. +Ns (= Eq+Ns & So+Ns) | medium | medium | **often** | **often** |
| 6. –Ns (= Eq-Ns & So-Ns) | **often** | **often** | **often** | medium |
| 7. Eq (= Eq+Ns & Eq-Ns) | **often** | **often** | **often** | **often** |
| 8. So (= So+Ns & So-Ns) | **often** | **often** | **often** | medium |
| 9. All (= Eq+Ns & So+Ns & Eq-Ns & So-Ns) | **often** | **often** | **often** | **often** |

3) Due to the new Table 5, subsection ‘3.5. Comparison of Viking navigators’ is modified as follows:

Table 4 compares the navigation success *S* of the three navigator types in nine different cases versus the four navigation methods. It is clear from Table 4 that the order number i of sailors slightly depends on the case number and method. Summarizing the essence of Table 4, Table 5 shows the best navigator type having order number i. in Table 4 with the largest averaged navigation success *S* in the 9 different cases studied versus the four navigation methods. ...

Hence, it is clear from Tables 4 and 5 that in most cases and for most navigation methods, the often-navigating sailor is the best (with order number i), that is this sailor type can navigate the ship with the highest sailing success.

**Reviewer 2 wrote**: *5. In Discussion section, please discuss your findings in the form of bullets. Currently, it is messed up.*

**Answer**: The revised ‘4. Discussion’ section is organized with the use of the following subsections:

4.1. Viking navigation

4.2. Navigation method and periodicity

4.3. Sailing date

4.4. Night sailing

Only subsections ‘4.1. Viking navigation’ and ‘4.2. Navigation method and periodicity’ are discussed in the form of several bullets, because in the other two subsections (4.3 and 4.4) there would be only one bullet point, which would not make sense, in our opinion.

**Reviewer 2 wrote**: *6. In Conclusion, please remove all bullets and describe in a paragraph and also add one more sentence for future work.*

**Answer**: The bullet form of the Conclusions section was suggested by one of the earlier Reviewers. Now, the bullets were removed, and the following sentence for future work was added to the end of this section:

An important future work could be the experimental field test of the sailing success of different possible Viking navigation methods onboard a research vessel crossing the Atlantic Ocean between Bergen (Norway) and South Greenland.

**Reviewer 2 wrote**: *7. In Methods, authors should include a pseudocode of their developed method. Currently, it is messed up and very hard for the reader to maintain flow. A pseudocode will give readers a fair insight about the developed algorithm.*

**Answer**: As suggested by Reviewer 2, to demonstrate qualitatively our developed computation/simulation method, the following new Figure 2 is inserted into subsection ‘2.2. Novelties of simulations’ of the revised ‘2. Methods’ section:

**Fig. 2. Block diagram of our developed computation/simulation of Viking navigation.**

**Reviewer 2 wrote**: *2. Contributions are clear.*

**Answer**: Thanks. :-)

**Reviewer 2 wrote**: *8. Overall, novelty seems high and write up is good. Authors must adjust my above changes.*

**Answer**: Thanks. :-) The above-suggested changes were done in the revised manuscript.

**Point-by-Point Response to the Comments of the Reviewer 3**

We thank Reviewer 3 for her/his valuable and constructive comments. Our manuscript was revised on the basis of the reports of three new Reviewers (1, 2, 3) and one old Reviewer (4). All changes suggested by Reviewer 1, Reviewer 2, Reviewer 3 and Reviewer 4 are marked with red, blue, orange and violet, respectively. Below is our response to the comments of Reviewer 3.

**Reviewer 3 wrote**: *The authors have addressed all issues and comments raised by reviewer 3. Therefore, I don't have further comments. The paper title is too long. This should be concisely described.*

**Answer**: The shortened new title suggested by Reviewer 4 is:

Speedy Bearings to Slacked Steering: Mapping the Navigation Patterns and Motions of Viking Voyages

**Reviewer 3 wrote**: *I would also like to suggest improving figure quality.*

**Answer**: The original resolution of the submitted tiff-format figures was high enough, but during the pdf-conversion the resolution became reduced. In the final published version all figures will have high resolution.

**Reviewer 3 wrote**: *The references are also quite old. Only one of the articles is referred from 2022.*

**Answer**: The research field of Viking navigation develops only slowly due to the only sporadic new data, information and archaeological findings. Since there are only a few scientists who study this subject, only very few publications appear yearly/decadely in this topic. This is the reason for the lack of many references from the last 5 years. Nevertheless, in the ‘Introduction’ section we cited the following 5 new, most recent references:

7. Vanin G. The beginning of celestial navigation. arXiv:2209.02371 2022. (https://doi.org/10.48550/arXiv.2209.02371).

13. Craecker-Dussart de C. Moyens d’orientation et de navigation des Vikings, marins accomplis en Atlantique Nord (fin VIII^e^–XI^e^ siècles). Le Moyen Age 2019; 3-4: 617-650 (doi: 10.3917/rma.253.0617).

38. Liu J, Zhao L, Qi B, Cheng J, Cai J. A new polar integrated alignment algorithm with the aids of DVL and the improved polarized-light navigation. Proc. 5th Int. Symp. Auto. Syst. (ISAS) 2022; 1-6 (doi.org/10.1109/ISAS55863.2022.9757316).

39. Courtier G, Lapray P-J, Adam R, Changey S, Lauffenburger J-P. Ground vehicle navigation based on the skylight polarization. IEEE/ION Position, Location and Navigation Symposium (PLANS) 2023: Monterey, United States. pp. 1373-1379 (doi: 10.1109/PLANS53410.2023.10140044).

40. Li Q, Dong L, Hu Y, Hao Q, Wang W, Cao J, Cheng Y. Polarimetry for bionic geolocation and navigation applications: a review. Remote Sensing 2023; 15: 3518 (doi: 10.3390/rs15143518).

Thus, together with the above 5 new references, in our revised manuscript there are 9 references from the last five (2019-2023) years including the followings:

10. Filipowiak W. How Vikings crossed the North Atlantic? The reinterpretation of ‘sun compasses’ - Narsarsuaq, Wolin, Truso. International Journal of Nautical Archaeology 2020; 49: 318-328 (doi: 10.1111/1095-9270.12426).

33. Takács P, Száz D, Pereszlényi Á, Horváth G. Sensitivity and robustness of sky-polarimetric Viking navigation: Sailing success is most sensitive to night sailing, navigation periodicity and sailing date, but robust against weather conditions. 2022; PLoS One 17: e0262762 (htpps://doi.org/10.1371/journal.pone.0262762)

36. Jin H, Wang X, Fan Z, Pan N. Linear solution method of solar position for polarized light navigation. IEEE Sensors Journal 2021; 21: 15042-15052 (doi: 10.1109/jsen.2021.3074416).

37. Dou Q, Du T, Qiu Z, Wang S, Yang J. An adaptive anti-disturbance navigation method for polarized skylight-based autonomous integrated navigation system. Measurement 2022; 202: 111847 (doi: 10.1016/j.measurement.2022.111847).

**If Reviewer 3 writes me the bibliographic data of such papers from 2019-2023, I shall add them to the reference list and cite them in our paper.**

**Point-by-Point Response to the Comments of the Reviewer 4**

We thank Reviewer 4 for her/his valuable and constructive comments. Our manuscript was revised on the basis of the reports of three new Reviewers (1, 2, 3) and one old Reviewer (4). All changes suggested by Reviewer 1, Reviewer 2, Reviewer 3 and Reviewer 4 are marked with red, blue, orange and violet, respectively. Below is our response to the comments of Reviewer 4.

**Reviewer 4 wrote**: *The authors have adequately revised their work as per the third and fourth reviewers' reports. Since the authors have requested the fourth reviewer to quote a paragraph on the Indian Ocean sailing techniques, they are given the permission to do so. The reference information on the said citation is as follow: Goswami, Chhaya. 2015 Malam Ni Pothi: Treatise on Kachchhi Navigation Techniques. Publisher- Darshak Itihas Nidhi, Mumbai. (Summarized transcription and a critique of the original sailing manuals written in old Gujarati.)*

**Answer**: Thank you for your kind permission and the bibliographic data of the suggested new reference. In the revised subsection ’4.1. Viking navigation’ of the ’4. Discussion’ section we wrote:

In one of the navigation methods of sailors crossing the Indian Ocean, at night the Polaris and/or the Moon served as time indicator (with the kind permission of the anonymous Reviewer 4) [36]: ”*In this strategy, ...*”

36. Goswami C. 2015; Malam Ni Pothi: treatise on Kachchhi navigation techniques. Darshak Itihas Nidhi Publisher: Mumbai, India (Summarized transcription and a critique of the original sailing manuals written in old Gujarati).

**Reviewer 4 wrote**: *The authors have mentioned that it is little known that how exactly the Vikings sailed in the night. On this I suggest them to consider a separate investigation based upon the cutting edge field research, consulting fragmented unconventional archives (log book or any such written accounts), oral narratives, to trace out or guesstimate the patterns and rhythms of their lunar sailings.*

**Answer**: This suggested investigation is indeed important and interesting, which should/could be performed in the future.

**Reviewer 4 wrote**: *I also have a suggestion for the authors regarding their long title, if they wish to change or modify accordingly. "Speedy Bearings to Slacked Steering: Mapping the Navigational Patterns and Motions of the Viking Voyages" Please note it is just a suggestion.*

**Answer**: Thank you very much for the suggested informative new title, which is accepted by us with pleasure. :-) Thus, the new shortened title of our revised manuscript is the following:

Speedy Bearings to Slacked Steering: Mapping the Navigation Patterns and Motions of Viking Voyages
